# Integrated single-cell and proteomic profiling of pancreatic neoplasia identifies tissue markers that outperform established diagnostic markers in distinguishing cancer from precursor lesions

**DOI:** 10.64898/2026.10.02.26364030

**Published:** 2026-10-07

**Authors:** Thoa T. Tran, Phuong-Uyen Nguyen-Hoang, Johannes F. Fahrmann, Duy Truong Pham, Huy Bui, Loan Bui, C. Max Schmidt

## Abstract

Pancreatic ductal adenocarcinoma (PDAC) arises within an extensively remodeled microenvironment, yet tissue markers that distinguish invasive carcinoma from precursor lesions remain limited. Integrating four single-cell RNA-sequencing atlases comprising 324,662 cells from 26 patients, we identified cancer-associated fibroblast expansion, accumulation of C1QA+ immunoregulatory macrophages, and depletion of a dendritic-cell compartment expressing plasmacytoid markers. Fibroblasts were enriched for extracellular-matrix and PI3K–AKT programs and macrophages for an antigen-presentation program. We then examined the protein-level correlates of these populations in a tissue proteomic reference framework of 356 samples spanning non-diseased pancreas, normal-adjacent tissue, low- and high-grade intraductal papillary mucinous neoplasms (IPMNs), IPMN-associated invasive carcinoma, and PDAC, prioritizing proteins that changed monotonically across the six histopathologic reference groups or marked tumor-enriched cell types. Despite depletion of plasmacytoid dendritic cells, the principal source of type I interferon, the interferon-stimulated protein ISG15, expressed by both fibroblasts and macrophages, increased monotonically toward PDAC. In exploratory cross-validated benchmarking of PDAC versus low- and high-grade IPMN, excluding IPMN-associated invasive carcinoma (145 versus 56 samples), CATB+EPIPL achieved an AUC of 0.98, with a sensitivity of 92% at an achieved specificity of 92%, exceeding protein-abundance models of diagnostic IHC-associated markers (S100P+IMP3+maspin+MSLN, 0.79) and refitted published IPMN-progression combinations (≤0.91). Against non-diseased pancreas, candidate and comparator panels performed similarly (AUC 0.99–1.00). These biologically informed tissue protein panels warrant independent validation, including immunohistochemical evaluation of EUS-guided fine-needle biopsy specimens and measurement in blood.

## Full Text Availability

The license terms selected by the author(s) for this preprint version do not permit archiving in PMC. The full text is available from the preprint server.

